# Changes in Anti-SARS-CoV-2 IgG Subclasses over Time and in Association with Disease Severity

**DOI:** 10.3390/v14050941

**Published:** 2022-04-29

**Authors:** Zoia R. Korobova, Elena V. Zueva, Natalia A. Arsentieva, Oleg K. Batsunov, Natalia E. Liubimova, Irina V. Khamitova, Raisa N. Kuznetsova, Artem A. Rubinstein, Tikhon V. Savin, Oksana V. Stanevich, Alexandr N. Kulikov, Dmitry E. Pevtsov, Areg A. Totolian

**Affiliations:** 1 Saint Petersburg Pasteur Institute, 14 Ulitsa Mira, 197101 St. Petersburg, Russia; elenazueva9@gmail.com (E.V.Z.); arsentieva_n.a@bk.ru (N.A.A.); batsunov@gmail.com (O.K.B.); natelu@mail.ru (N.E.L.); div-o@mail.ru (I.V.K.); kuznetzova.rais@yandex.ru (R.N.K.); savintihon@gmail.com (T.V.S.); totolian@spbraaci.ru (A.A.T.); 2 Department of Immunology, Department of Infectious Diseases, Intensive Care Unit, Pavlov First State Medical University of St. Petersburg, 6-8 Ulitsa L’va Tolstovo, 197022 St. Petersburg, Russia; arrubin6@mail.ru (A.A.R.); oksana.stanevich@gmail.com (O.V.S.); ankulikov2005@yandex.ru (A.N.K.); dmitriipevtcov@gmail.com (D.E.P.)

**Keywords:** COVID-19, IgG, IgG subclasses, severity

## Abstract

IgG is the most prominent marker of post-COVID-19 immunity. Not only does this subtype mark the late stages of infection, but it also stays in the body for a timespan of at least 6 months. However, different IgG subclasses have different properties, and their roles in specific anti-COVID-19 responses have yet to be determined. We assessed the concentrations of IgG1, IgG2, IgG3, and IgG4 against different SARS-CoV-2 antigens (N protein, S protein RBD) using a specifically designed method and samples from 348 COVID-19 patients. We noted a statistically significant association between severity of COVID-19 infection and IgG concentrations (both total and subclasses). When assessing anti-N protein and anti-RBD IgG subclasses, we noted the importance of IgG3 as a subclass. Since it is often associated with early antiviral response, we presumed that the IgG3 subclass is the first high-affinity IgG antibody to be produced during COVID-19 infection.

## 1. Introduction

Since late December 2019, the world has been facing a new threat from severe acute respiratory syndrome-coronavirus-2 (SARS-CoV-2) [1]. The pandemic has caused the deaths of over six million people as of March 2022. Even with the introduction of various vaccines, it is still as present in the human population as it was in the year 2020 [2]. According to a Cochrane review of 16 COVID-19-related studies (representing over 7000 patients), COVID-19 infection is usually associated with a variety of symptoms: cough; sore throat; fever; and musculoskeletal symptoms, such as myalgia, fatigue, or headache [3]. Among clinical signs that can be detected via diagnostic imaging (chest radiography, CT, ultrasound), there are specific COVID-19-induced lung tissue changes, including ground glass opacities and fibrosis-like morphology [4].

There is, however, a relatively small stratum of patients who do not develop any clinical symptoms up to the point when SARS-CoV-2 RNA is an incidental finding during routine PCR testing of samples [5]. In some cases, in relatively healthy patients with no known history of COVID-19, SARS-CoV-2-specific immunoglobulins of subtype G (IgG) can be found [6].

One of the most often used markers for assessment of post-COVID-19 immunity is serum antibody (Ab) concentration. Although B-cell immunity is not the driving force in antiviral responses, its effector molecules can still be used as markers of sufficient immunity, in terms of both post-infection and post-vaccination responses [7]. In COVID-19-associated immunity, out of five Ig allotypes (A, M, G, E, D), only IgA, M, and G are involved in immune response, with IgG, specifically, being the most abundant [8].

Among existing ELISA kits for assessment of SARS-CoV-2 immunity, there are both quantitative and qualitative kits. They are based on reactivity with different parts of the virion, or its various antigens: spike protein (S protein); S protein receptor-binding domain (RBD); or nucleocapsid protein (N protein). Quantitative and qualitative factors influence the diagnostic accuracy of such tests according to a systematic review of 40 studies (representing over 29,000 tests) [9]. According to the same review, the sensitivity of such kits also relies on timing: detection by ELISA is significantly lower in patients in the first week of the infection onset, with significant improvement in detection starting in the second week.

Among all anti-SARS-CoV-2-specific antibodies, the IgG subtype is the most prominent marker of post-infectious immunity. Not only does this subtype mark the late stages of infection, but it also stays in the body for a period of at least 6 months [10]. However, there are different IgG subclasses with different properties (IgG1, IgG2, IgG3, IgG4) [11]. Their roles in the anti-COVID-19-specific response have yet to be determined. In our research, we assessed specific anti-SARS-CoV-2 IgG subclasses in patients, relative to COVID-19 severity and the stage of illness.

## 2. Materials and Methods

### 2.1. Cohort Description

Plasma samples were taken from 348 patients, aged 18 to 85 years, who were 45.11% female (*n* = 157) and 54.89% male (*n* = 191). The stage of COVID-19 infection varied from day 1 of clinical manifestation (and/or first positive PCR result) to 438 days since an officially registered COVID-19 infection. Among included patients: 10.06% (*n* = 32) were infected with COVID-19 at the time of blood collection; 47.48% (*n* = 151) were considered ‘early’ convalescents (samples taken < 6 mo after COVID-19 onset); and 42.45% (*n* = 135) were considered ‘late’ convalescents (> 6 mo since the onset). None of the patients (acute or convalescent) were vaccinated prior to our study. No known cases of COVID-19 reinfection in patients within the cohort occurred prior to sampling. All samples were collected between April 2020 and July 2021. Samples collected in the first half of 2020 included individuals sick at the time of sample collection (Wuhan strain was prominent at that time). Subsequent collection was from convalescent individuals and featured other emergent strains.

COVID-19 severity in patients varied. Out of 348 patients, the situation was as follows: 22.10% (*n* = 77) presented with mild illness; 33.62% (*n* = 117) presented with moderate severity; and 21.83% (*n* = 76) presented with severe infection. In 22.41% (*n* = 78) of cases, we could not establish infection severity due to a lack of clinical information provided with the samples. Patients with moderate to severe illness were treated at Pavlov First Saint Petersburg State Medical University, a COVID-19-specialized hospital. Patients with mild severity were treated at home or at local clinics in Saint Petersburg. ‘Early’ and ‘late’ convalescents included in the study were patients of the Saint Petersburg Pasteur Institute Medical Centre (Saint Petersburg, Russia).

Severity assessment was performed by the hospital medical staff, after patient discharge, and was based on the Russian Ministry of Health guidelines. To estimate cut-off values for ELISA, we used samples from 48 healthy donors with no prior history of COVID-19 or vaccination. Their age (Me ± SD) was 55 ± 18, with 66.7% females (*n* = 32) and 33.3% males (*n* = 16). All demographical information on both cohorts is provided in Table 1.

The study’s protocol was approved by the ethics committee of the Saint Petersburg Pasteur Institute in accordance with the Declaration of Helsinki. All participants were informed of our study and willingly signed consent forms.

### 2.2. Sample Preparation

Peripheral blood was used as study material. Blood samples were collected in vacuum tubes with EDTA anticoagulant, followed by centrifugation (350 g for 10 min). Plasma samples were transferred to cryotubes and frozen at −80 °C before ELISA.

### 2.3. Methodology

Commercial ELISA kits were used to assess total specific IgG against N protein and S protein receptor-binding domain.

### 2.4. Quantitative Assessment of Anti-N Protein IgG

We used a commercial ELISA kit to assess total specific IgG against the SARS-CoV-2 N protein, designed and manufactured by the Saint Petersburg Pasteur Institute (registered for commercial use with the Federal Service for Surveillance in Healthcare (Roszdravnadzor, date of registration 14.02.2021)). All ELISA protocols were performed according to manufacturer’s instructions. Prior to analysis, we diluted the conjugate in PBS IHC Wash Buffer (1:50) and prepared calibrators and controls by adding them to the assigned wells in the required concentrations. Pre-diluted (1:100) samples were then added. Incubation with buffer for this step was 1 h at 37 °C. The plate was then washed 3 times with buffer and conjugate added (100 μL/well), with subsequent incubation (1 h, 37 °C). After a final washing step, TMB (100 μL/well) was added, followed by incubation for 10 min in darkness. After incubation, stop reagent (50 μL/well) was added and results were registered. A calibration curve was made according to the instructions (Table 2). The cut-off value established for this kit was 16.95 BAU/mL.

### 2.5. Quantitative Assessment of Anti-S Protein IgG

For analysis of Abs to S protein RBD, the quantitative ELISA kit manufactured by LabPack LLC was used (Saint Petersburg, Russia, date of registration 16 September 2021). Prior to analysis, samples were diluted 1:100. Calibrators and conjugates were prepared as directed by the manufacturer. Samples and calibrators were incubated in the plate with buffer for 30 min with agitation (650 rpm, 37 °C). After aspiration, the remaining well contents were washed 5 times with wash buffer. Conjugate (100 μL/well) was added shortly after, followed by incubation for 30 min. After incubation, wells were washed 5 times, TMB substrate was added, and the plate was then incubated for 10 min in darkness. Stop reagent was then added to terminate the reaction. The cut-off value established for this kit was 22.6 BAU/mL.

### 2.6. Analysis by Subclass

For our intended analysis, we designed two specific ELISA methodologies to assess subclasses of IgG against different SARS-CoV-2 antigens (N protein, S protein RBD). Both methods included reagents for detection of each IgG subclass (IgG1, IgG2, IgG3, IgG4).

### 2.7. Quantitative Assessment by Anti-N Protein IgG Subclass

For detection of anti-N protein Ab subclasses, we diluted samples differently for each subclass: IgG1 (1:100); IgG2 (1:25); IgG3 (1:25); and IgG4 (1:100). The diluted samples and pre-added standards of different concentrations were incubated (30 min, 37 °C), then washed 4 times with PBS IHC Wash Buffer, before incubation with conjugate. Prior to adding TMB substrate (100 μL/well), the plate was washed again. Stop reagent was used to terminate reaction (50 μL/well). The calibration curve and determinations were calculated as BAU/mL. Calibration reference values are presented in Table 3. Raw concentration determinations were later multiplied by the dilution factor, 25 or 100, noted above.

To calculate cut-off limits, values from samples known to be seronegative (total IgG against N protein) were used. These samples belonged to healthy donors (*n* = 48) with no previous history of COVID-19. The cut-off values are presented in Table 4.

For technical reasons, only 312 out of the 348 samples were available for assessment of anti-S protein IgG. Prior to analyzing anti-S protein Ab subclasses, samples were diluted 1:50. The samples and pre-added standards of different concentrations were incubated in buffer (30 min, 37 °C), followed by 4 washes with PBS IHC Wash Buffer. The conjugate was added (100 μL/well) and allowed to incubate (30 min), followed by 4 plate washes. TMB (100 μL/well) was then added and left to react for 10 min (37 °C). Reactions were terminated with stop reagent (50 μL/well). The calibration curve and determinations were calculated as BAU/mL. Calibration reference values are presented in Table 5, and cut-off values are presented in Table 6.

We used experimental ELISA kits designed and manufactured specifically for this study. This puts a serious limitation on our study: the cut-off values used were established based on both the calibration curve and samples that were seronegative for total IgG (quantitative ELISA).

### 2.8. ELISA Readings

All ELISA results were registered using the Multiskan FC Microplate Photometer (Thermo Fisher Scientific, Waltham, MA, USA). Optical densities were registered in two modes: 450 nm and 620 nm for quantitative kits (anti-N protein and anti-S protein total IgG) and 450 nm and 520 nm for subclasses.

### 2.9. Statistical Analysis and Visualization

For comparison of multiple data groups, the Kruskal–Wallis test was used. For two-group analysis, we used the Mann–Whitney *U*-test for continuous variables, and the χ2 Fisher’s test for categorical variables. Correlation analysis of ELISA results was performed via the Spearman correlation coefficient (rs). When interpreting correlation analyses, we interpreted rs values as follows: 0.2–0.39 as weak correlation; 0.4–0.69 as moderate; 0.7–1.0 as strong. When interpreting statistical analysis results, we designated *p* < 0.05 to be statistically significant.

Data visualization was performed via Graphpad Prism 8.0 and Microsoft Excel 2013 graphing tools. To represent antibody concentration dynamics (trends), polynomial graphing in Microsoft Excel was used. For visualization of statistical differences in COVID-19 severity, Graphpad Prism 8.0 was used. All statistical analysis was performed in Graphpad Prism 8.0.

## 3. Results

### 3.1. Antibodies against SARS-CoV-2 N Protein

Out of 348 samples, 100 (28.70%) were considered seronegative for anti-N protein antibody (IgG < 17 BAU/mL as per the manufacturer’s instructions). Among seronegative samples, illness severity was mild in 30 cases (30.00%), moderate in 16 cases (16.00%), and severe in 9 cases (9.00%). In 20 cases (20.00%), severity was not established due to a lack of clinical data provided with the samples.

**Figure 1:**
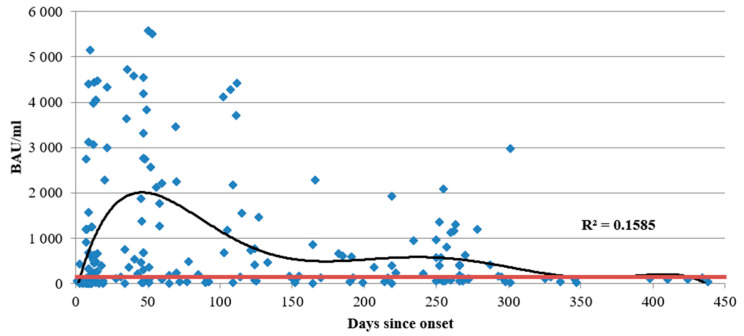
Total anti-N protein antibody dynamics (quantitative ELISA) as a function of time since COVID-19 onset for 348 individual patients. The range was from 1 to 438 days. IgG concentration is presented in BAU/mL. The red dotted line represents the cut-off value for this specific ELISA kit (17 BAU/mL). The black line is the trend (r^2^ = 0.16).

**Figure 2:**
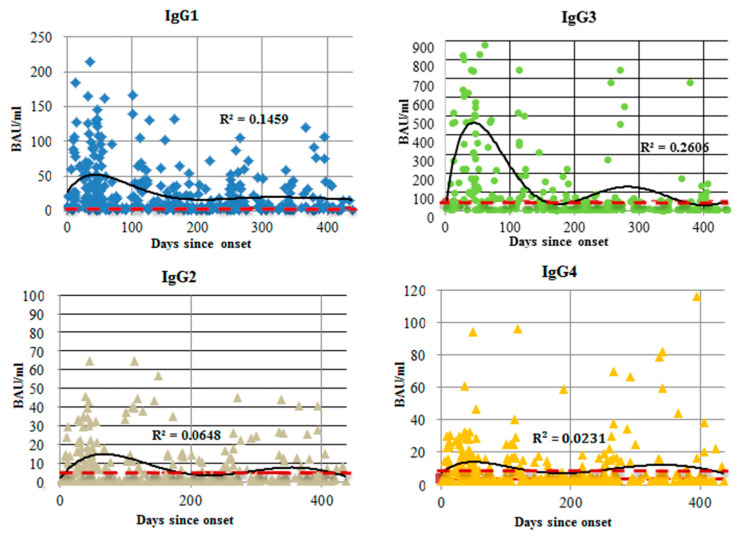
Total anti-N protein IgG antibody dynamics (ELISA) by subclass, as a function of time since COVID-19 onset for 348 individual patients. The range was from 1 to 438 days. Concentration is presented in BAU/mL. The red line represents the cut-off value for this specific subclass. The black line is the trend, and R^2^ value is shown on each graph.

Out of all seronegative samples, 35 (35.00%) were from patients in the acute phase of SARS-CoV-2 infection (within 14 days of onset). In 20% of seronegative cases, sample collection occurred within an intermediate interval after onset (from 15 days up to 6 months). A significant portion (*n* = 45, 45.00%) of seronegative samples was from patients with COVID-19 more than 6 months prior to sample collection. Figure 1 shows the total anti-N protein IgG concentration dynamics as a function of time since COVID-19 onset. Figure 2 shows the changes in anti-N protein IgG subclass over time.

**Figure 3:**
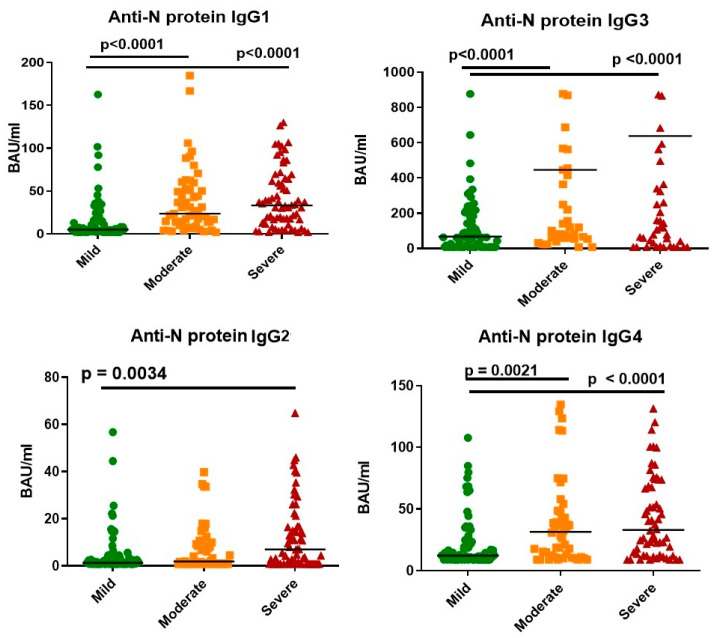
Differences in anti-N protein IgG subclass concentration depending on illness severity, Me (Q25–Q75).

**Figure 4:**
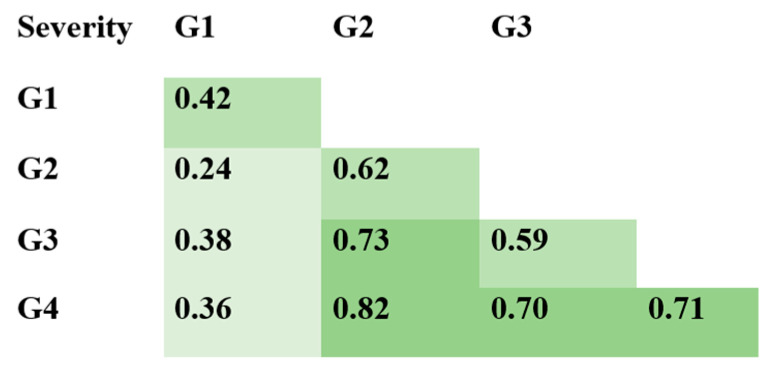
Correlation matrix for anti-N protein IgG subclasses and illness severity, analyzed by Spearman correlation coefficient. Darker shades of green represent more solid correlation: 0.2–0.39—weak correlation; 0.4–0.69—fair correlation; 0.7–1.0—solid correlation. *p*-value for all presented coefficients < 0.0001.

We also examined the relationships between anti-N protein Ab subclasses and illness severity (Figure 3 and Figure 4). In general, there were statistically significant differences in IgG concentration between mild and more severe infection. Correlation analysis (Figure 4) showed a positive correlation between subclass concentrations and illness severity.

### 3.2. Antibodies against SARS-CoV-2 S Protein

**Figure 5:**
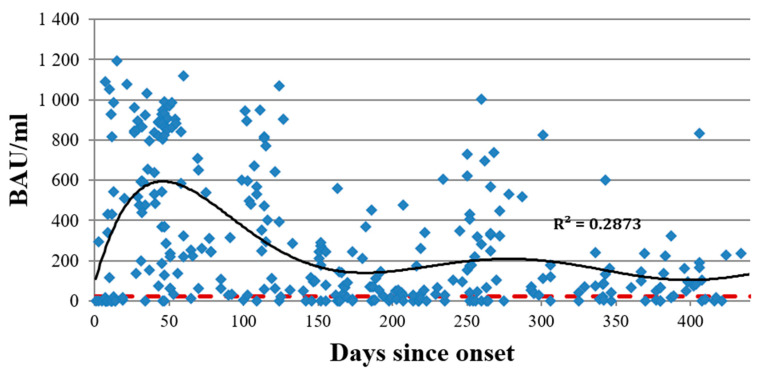
Total anti-S protein (RBD) antibody dynamics (ELISA) as a function of time since COVID-19 onset for 312 individual patients. The range was from 1 to 438 days. IgG concentration is presented in BAU/mL. The red dotted line represents the cut-off value for this specific ELISA kit (22.6 BAU/mL). The black line is the trend, with an R^2^ of 0.28.

For technical reasons, only 312 samples were available for assessment of the anti-S protein IgG. Of them, 71 (22.75%) were negative for anti-S protein antibody (cut-off of 22.6 BAU/mL). Of these serologically negative samples, 16 samples (22.50%) were from patients in the acute phase of illness (within 14 days of onset). In 21% of seronegative cases, sample collection occurred within an intermediate interval after onset (from 15 days up to 6 months). Forty samples (56.30%) were from patients with COVID-19 more than 6 months prior to sample collection. The results are presented in Figure 5.

**Figure 6:**
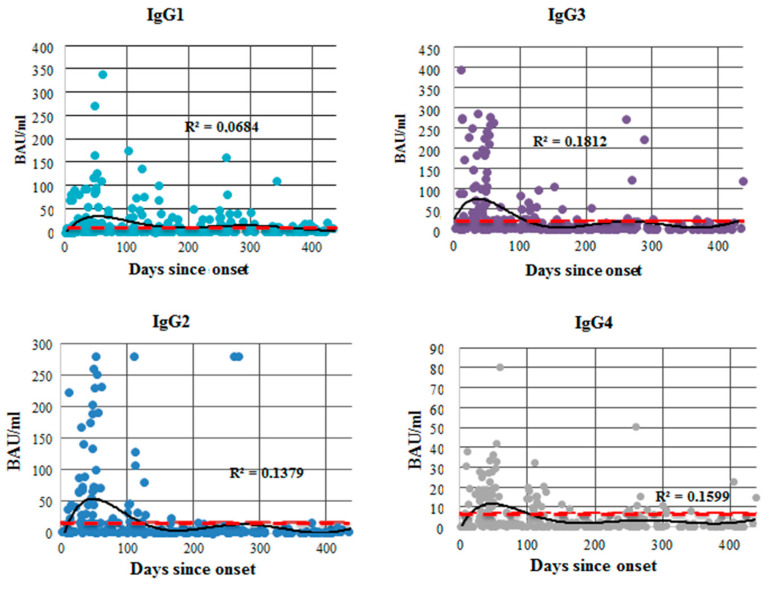
Total anti-S protein (RBD) IgG antibody dynamics (ELISA) by subclass, as a function of time since COVID-19 onset for 312 individual patients. The range was from 1 to 438 days. Concentration is presented in BAU/mL. The red line represents the cut-off value for this specific ELISA kit. The black line is the trend. R^2^ values are shown on the graph.

After assessing total anti-S protein (RBD) Ab concentrations, we measured concentrations of each IgG subclass in all 312 samples. The results are presented in Figure 6. Peaks in concentration were generally seen within the first 100 days of illness.

**Figure 7:**
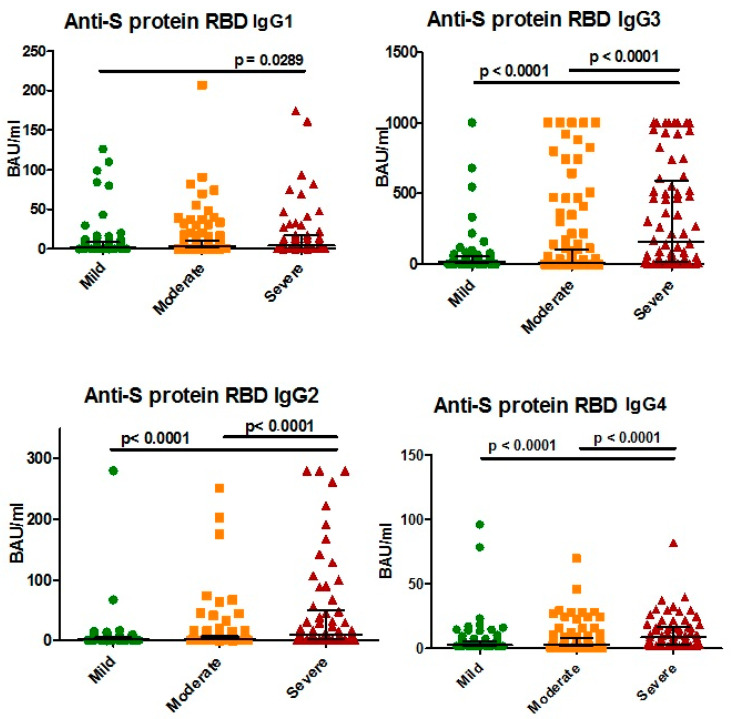
Differences in anti-S protein (RBD) IgG subclass concentration depending on illness severity, Me (Q25–Q75).

**Figure 8:**
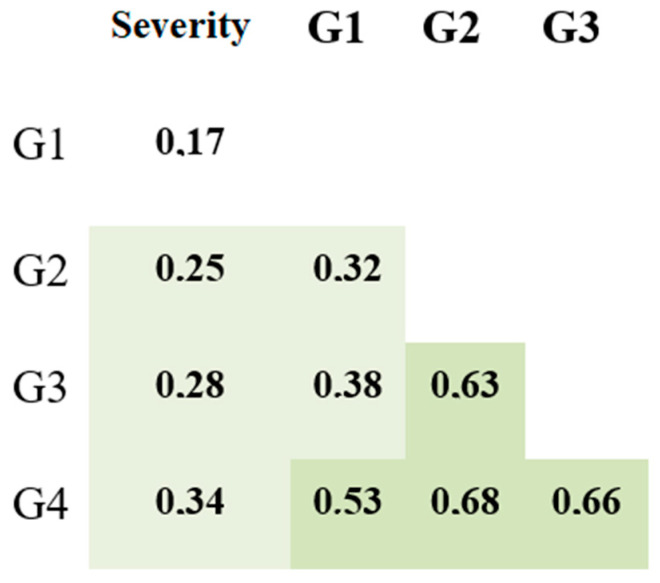
Correlation matrix for anti-S protein IgG subclasses and illness severity, analyzed with Spearman correlation coefficient. Darker shades of green represent more solid correlation: 0.2–0.39—weak correlation; 0.4–0.69—fair correlation; 0.7–1.0—solid correlation. *p*-value for all presented coefficients < 0.001.

We also compared anti-S protein Ab concentrations relative to illness severity and performed correlation analysis (Figure 7 and Figure 8). In general, there was a statistically significant difference in IgG concentration between mild and severe cases of infection. Correlation analysis in Figure 4 showed a positive correlation between subclass concentrations and illness severity.

## 4. Discussion

### 4.1. Total Anti-SARS-CoV-2 IgG Assessment

When assessing both anti-N protein and anti-S protein antibodies (Abs), we noticed a group of so-called ‘seronegative’ samples. Although all patients who donated blood in this study had been previously diagnosed with COVID-19, there was a portion of samples with IgG levels below established cut-off values.

Regarding anti-N protein Abs, these samples were taken from patients in different stages of illness; 35.00% were collected from patients within the first 14 days of infection onset. This finding is supported by a review of 143 studies published by Colombian researchers in 2021, indicating that seroconversion from IgM to IgG generally happens in the time period between the 12th and 14th day of SARS-CoV-2 infection [12]. A significant portion (45.00%) of seronegative samples here belonged to patients who had COVID-19 more than 6 months prior to sample collection. The period between the 3rd and 6th month since illness onset is mentioned in multiple works concerning declining IgG levels [13,14,15,16].

When assessing anti-S protein Abs, we found 71 samples (22.75%) to be negative. Of these, 16 samples (22.50%) were taken from patients in the acute stage of illness (within the first 14 days of onset), and 40 samples (56.30%) were taken more than 6 months since onset.

For analysis, we designated samples collected from 2 weeks to 6 months after onset of illness as ‘intermediate collection’ samples. In 20% (*n* = 20) of anti-N protein seronegative cases, sample collection occurred in the intermediate time frame; this is 5.7% of the entire tested cohort (*n* = 348). In 21% (*n* = 15) of anti-S protein seronegative cases, sample collection occurred in the intermediate time frame; this is 4.8% of the entire tested cohort (*n* = 312).

Only 33 samples (9.65%) were negative for anti-S protein and anti-N protein antibodies: 11 samples (33.33%) were taken within the first 2 weeks after COVID-19 manifestation; 18 (54.54%) were taken after the 6th month. These data correlate with information provided by researchers from various countries and continents: among all cases of SARS-CoV-2 infection, up to 10% of people feature inadequate Ab production [17,18,19,20].

### 4.2. Anti-N Protein IgG Subclasses

When comparing anti-N protein Ab concentrations by IgG subclass, we noted two points. Firstly, there was a correlation between illness severity and IgG subclass concentration. The Spearman’s rank correlation (rs) for IgG1, IgG2, IgG3, and IgG4 were 0.42, 0.21, 0.37, and 0.36, respectively (*p* < 0.0001). We considered this correlation to be weak. Similar findings (correlation between Ab concentration and illness severity) have been presented in multiple studies [21,22,23,24,25], although in those studies the main focus was on total anti-N protein IgG levels.

Secondly, among all four subclasses of anti-N protein IgG, IgG3 showed the most prominent concentration dynamics in response to COVID-19. This may be due to the specific structure or function of IgG3. Among all existing subclasses, IgG3 (along with IgG1) was the most involved in antiviral response, and was claimed to be the first subclass to appear during infection [26]. This Ab subclass is partially responsible for mediation of effector functions in inflammation [11]. Although all four IgG subclasses have nearly 90% structural similarity, IgG3 has the longest hinge region. It contains up to 62 amino acids (including 21 proline and 11 cysteine residues), forming a poly-proline helix with limited flexibility. Due to this factor, which affects its ability to move and bind antigen, as well as its molecular mass, IgG3 is often described as the subclass with the highest binding capacity [27,28]. As such, the role of IgG3 in COVID-19 development and pathogenesis merits further clarification, potentially as a marker of severity or outcome.

### 4.3. Anti-S Protein IgG Subclasses

Like anti-N protein IgG, total anti-RBD IgG showed a tendency to remain in the body up to 500 days after the onset of illness at levels higher than the cut-off value. However, when assessing anti-S protein (RBD) IgG subclasses, we noted dynamics that slightly differed from those seen with anti-N protein Abs. IgG1-G4 concentrations had a weak correlation with the COVID-19 severity: rs of 0.17, 0.25, 0.28, and 0.36 for G1, G2, G3, and G4, respectively (*p* < 0.001). We also saw that in patients with mild COVID-19, all four subclasses were significantly lower in concentration compared to those in more severe forms of the infection. Notably, IgG1 had the weakest correlation with COVID-19 severity (r_s_ = 0.17), and it featured the smallest differences between severity groups (Figure 7).

Out of the four subclasses, IgG3 was more associated with initial illness (1–25 days, *p* < 0.05), whereas IgG1 and IgG2 concentrations tended to show an upsurge (*p* < 0.01 and *p* < 0.05, respectively) in the later stages and post-recovery period (180^+^ days). This may indicate a switch in subclass prevalence over time.

In samples from patients with severe infections, Ab concentrations were significantly higher than in those from patients with milder courses of illness. These data correspond with information provided by multiple sources [8,29,30,31,32]. Among all Ab subclasses analyzed (anti-S protein RBD), IgG4 had significantly lower concentrations compared to the other subclasses (*p* < 0.0001).

## 5. Conclusions

When analyzing IgG levels by subclass against different antigens (N protein, S protein RBD), we discovered a statistically significant correlation between COVID-19 severity and IgG concentrations (both overall and by subclass). Therefore, based on our findings, as well as findings from other researchers, we can safely assume that IgG levels are higher in patients with severe clinical presentation.

When assessing anti-S protein (RBD) antibody subclasses, we observed fluctuations in subclass persistence over the course of illness. IgG3 levels fell, whereas IgG1 and IgG2 levels were seen to rise over time. Among all anti-S protein Ab subclasses, IgG1 showed the weakest correlation with illness severity. We note the importance of IgG3 as a subclass. Since it is often associated with early antiviral response, we presume that the IgG3 subclass is the first IgG antibody of high-affinity to be produced during SARS-CoV-2 infection. It is also known to be the subclass with the highest binding capacity.

## Figures and Tables

**Table 1:** Demographics of study participants.

	COVID-19 Patients and Convalescents	Healthy Donors for Cut-Off Estimation
Number of samples	348	48
Age (Me ± SD)	62 ± 14	55 ± 18
Sex differences	45.11% female (*n* = 157) 54.89% male (*n* = 191)	66.7% female (*n* = 32) 33.3% male (*n* = 16)

**Table 2:** Calibrator values for the anti-N protein IgG kit.

	Antibody Concentration (BAU/mL)
Calibrator 1	10.16
Calibrator 2	5.08
Calibrator 3	2.54
Calibrator 4	1.27
Calibrator 5	0.63
Calibrator 6	0.31
Calibrator 7	0.15

**Table 3:** Calibrator values for each anti-N protein antibody subclass.

	Antibody Concentration (BAU/mL)			
	IgG1	IgG2	IgG3	IgG4
Calibrator 1	8.70	16.32	40.00	8.70
Calibrator 2	4.35	8.16	20.00	4.35
Calibrator 3	2.17	4.08	10.00	2.17
Calibrator 4	1.09	2.04	5.00	1.09
Calibrator 5	0.55	1.02	2.50	0.55
Calibrator 6	0.10	0.10	0.10	0.10

**Table 4:** Cut-off values for anti-N protein antibodies, calculated for each subclass.

Antibody Concentration (BAU/mL)				
IgG1	IgG2	IgG3	IgG4	
3.58		5.02	53.28	3.51

Quantitative assessment by anti-S protein IgG subclass.

**Table 5:** Calibrator values for each anti-S protein (RBD) antibody subclass.

	Antibody Concentration (BAU/mL)			
	IgG1	IgG2	IgG3	IgG4
Calibrator 1	340.00	500.00	395.00	385.00
Calibrator 2	250.00	280.00	245.00	255.00
Calibrator 3	95.00	100.00	115.00	100.00
Calibrator 4	45.00	32.50	65.00	50.00
Calibrator 5	20.00	12.50	35.00	20.00
Calibrator 6	8.00	5.00	20.00	10.00
Calibrator 7	3.30	2.25	10.00	5.00
Calibrator 0	0.00	0.00	0.00	0.00

**Table 6:** Cut-off values for anti-S protein (RBD) antibodies calculated for each subclass.

Antibody Concentration (BAU/mL)				
IgG1	IgG2	IgG3	IgG4	
9.45		16.98	20.00	7.00

## Data Availability

Not applicable.
